# Strategy and additive technologies as the catalyst for outsourcing, process innovation and operational effectiveness

**DOI:** 10.1371/journal.pone.0282366

**Published:** 2023-02-27

**Authors:** Thomas Tegethoff, Ricardo Santa, Edgardo Cayón, Annibal Scavarda

**Affiliations:** 1 Colegio de Estudios Superiores de Administración–CESA, Bogotá, Colombia; 2 Federal University of the State of Rio de Janeiro, Rio de Janeiro, Brazil; University of Salento, ITALY

## Abstract

**Purpose:**

There is rising interest in Industry 4.0 as a factor in the competitiveness of the organization. Although many companies are aware of the importance of Industry 4.0, the development of such initiatives in Colombia is slow. Consequently, this research investigates the impact of additive technologies as part of the Industry 4.0 concept on operational effectiveness and, therefore, the competitiveness of the organization and tries to establish the factors that hinder the adequate implementation of such new, innovative technologies.

**Design/Methodology/Approach:**

Structural equation modeling was used to analyze the antecedents and outcomes of operational effectiveness. To this end, 946 usable questionnaires were collected from managers and personnel from Colombian organizations.

**Findings:**

Initial findings show that management is aware of Industry 4.0 concepts and implements strategies for such initiatives. Nevertheless, neither process innovation nor additive technologies have a significant impact on operational effectiveness and therefore on the competitiveness of the organization.

**Practical implications:**

The implementation of new innovative technologies requires the closure of the digital gap between urban and rural areas and between large and medium and small enterprises. Similarly, the concept of Industry 4.0 as a new, innovative manufacturing concept requires a transversal implementation to increase the competitiveness of the organization.

**Originality/Value:**

The value of this paper lies in discussing the current technological and human capabilities and strategies that Colombian organizations, as an example of a developing nation, should improve to leverage the benefits of Industry 4.0 to remain competitive. The results are probably generalizable to other regions in developing countries throughout the world.

## 1. Introduction

International trade has a significant impact on the well-being of any society [1]. Consequently, it is necessary to enhance the competitiveness of the organization to survive in a constantly changing environment, thus adding to the well-being of the country and the society.

Germany, as a country relying heavily on international trade, decided to launch a country-wide initiative called *Industrie 4*.*0* (Industry 4.0) in 2011. The main characteristic of the concept of Industry 4.0 is the individualization of the product according to the customers’ requirements, but also within a flexible large-scale production. This kind of strategy has been defined as differentiation and is one of two generic strategies that enhances the competitive advantage of any organization [2, 3]. The production processes involve the direct participation of all groups of interest (starting with the supplier and business partners) in the whole value chain and the supply chain. The production chain requires the real-time integration of all production resources, such as people, machines, systems, and logistics, and direct cooperation between all of them. As a consequence, intelligent networks should not only optimize one part of the process but the whole production process [4–6].

Nowadays, Industry 4.0 is a global concept applied to most industrial and financial sectors. Industry 4.0 comprises the themes of the internet of things (integrated connectivity of systems, services, and objects), cloud computing (huge capacity of storage and instant access to the data), autonomous robots (interconnected robots within the production process), big data (a large amount of data analyzed by automatic systems), internet of services (services available on demand), augmented reality (virtual elements integrated into reality), cyber-physical system and human-digital interfaces (integration of humans and machines), additive manufacturing/3D printing (additive manufacturing processes), smart factory (manufacturing on the internet of things and cyber-physical systems), and blockchain (a decentralized database with full traceability). Such innovative changes in the manufacturing processes also require innovative changes in the organizations’ processes and organizational strategy to enhance competitiveness and operational effectiveness [7–9]. It also changes the strategic view of the organization in their process of implementation and the interaction with suppliers and customers [10, 11].

The concept of Industry 4.0 is also recognized in Colombia. According to a survey from 2019 by the National Association of Businessmen of Colombia (ANDI), 88.2% of their members know what the 4th industrial revolution is, and 65,2% have already designed a strategy for digital transformation. Additionally, the geography and the lack of adequate infrastructure on roads and highways in Colombia should be an incentive for the implementation of decentralized manufacturing processes, such as additive manufacturing, combined with the internet and automated processes, which also enhances the sustainable competitiveness of organizations. Nevertheless, the results are not encouraging [12].

Considering the novelty of Industry 4.0 in the country, and the importance of competitiveness in the social, economic, and political development of the country, the research question for this study is:

“What are the factors from Industry 4.0, that promote the operational effectiveness of local organizations”

## 2. Literature review

### 2.1 Operational effectiveness

Porter [2] proposes 2 generic strategies, that organizations should embrace to generate competitive advantage—differentiation and cost leadership. Operational effectiveness is part of the cost leadership strategy as it comprises a more efficient use of the resources of the organization. Operational effectiveness considers the dimensions of cost, quality, flexibility, speed, and reliability [13, 14].

Cost: The main aspect of cost contemplates the reduction of inefficiencies and waste within the production process [15, 16]. The reduction of cost implies changes in technologies and processes to avoid unnecessary expenses and a cost-benefit analysis is required when implementing such changes or acquiring new technologies [17, 18]. Cost performance also refers to the relationship between the cost of the changes or new technologies compared to organizational objectives and achieving the best correlation [19, 20].

Quality: The main concept of quality can be described as giving the customer what they want and what they need [16]. Nevertheless, there are many definitions of quality based on objective reasons or subjective perception. Manufacturing-based quality centers on the objective defect rate of the product or service. The product-based definition of quality refers to the attributes or characteristics of the service or product. User-based quality refers to what extent the product or service fulfills the user´s requirements or satisfies customers’ needs. Value-based quality includes not only the user´s requirements but also the price. In this context, the concept includes an acceptable price for the products or services [21]. The definitions also include the concept of service methods, response time, post-sale service, delay time, guarantee time, delivery time, service consistency, repair quality, responsible attitude, and service facilities and locations [22]. Consequently, to satisfy the customer´s requirements, the organization requires a culture of quality to enhance competitiveness [23].

Agility: The concept of agility is intrinsically linked to speed and flexibility. Speed for an organization is the time required to respond to customers’ requirements or the time necessary to develop and deliver a new product or service [14, 24, 25]. Consequently, speed is the ability to perform any process or action by the organization in the shortest possible timeframe [26]. Flexibility is intrinsically linked to the concept of speed, as it includes not only the response time to the client´s requirements but also eventual short-term changes in such requirements [13, 27, 28]. Therefore, agility can be defined as a multidimensional aspect that includes the ability to anticipate, understand, and react to any market change and thus involves the capability to adapt and to take advantage of those market opportunities with innovative solutions. The responses are based on the magnitude of the changes (flexibility) and the rate of variety (speed) that the organization can generate. Agility is the combination of high rates of speed and high rates of flexibility [29, 30].

Reliability: The concept of reliability is a core principle in customer satisfaction. Within the manufacturing or service environment, any product or service should function according to the provider’s description, including the promised function and minimum usage timeframe [31–33]. A similar definition is given by Kuo and Zuo [34] who propose that reliability can be defined as the condition that the product will not fail in the specified time frame and works as designed initially. It is important to note that the literature states that reliability is either acceptable or not acceptable and does not use the terms “high” or “low” reliability [35]. Reliability can be defined as product reliability or as process reliability. The first is product-based and considers the customer´s perception. The latter considers whether the organizational processes perform as expected and specified within certain parameters and norms. To summarize, reliability is the prediction of the probability that something will perform as specified, under the specified conditions, for the specified period [35].

### 2.2 Strategy

The concept of strategy is widely discussed within the academic world. Mintzberg [36] integrates five definitions of strategy: strategy as a plan, strategy as a pattern, strategy about what, strategy as a position, and finally, strategy as a perspective. He proposes that strategy is a shared abstract concept within the minds of the members of the organization. Consequently, strategy cannot be determined by analyzing the thoughts of one individual, but by observing the same behavior or thinking of many coworkers. Thus, strategy is not only a notion of how to deal with competitors in the market. It also penetrates some of the most fundamental issues about organizations as instruments for collective perceptions and actions [36]. Araujo and Easton [37] took Mintzberg´s definition and have gone one step further in proposing that strategy (based on strategy as a pattern) is a set of several actions with a certain degree of consistency within a certain timeframe.

On the other hand, strategy is an organizational concept that cannot be mistaken for operational effectiveness. Both are necessary to enhance competitive advantage and better performance. Operational effectiveness includes performing similar activities better than competitors. Strategy, on the other hand, includes developing activities that are different from those of the competitors or performing the same activities, but differently [2]. The basis of an adequate strategy is implementing unique activities, that cannot be easily matched by competitors. The environment and context of the organization are the sources of such unique activities.

Consequently, considering strategy as a pattern and as performing unique activities, two generic strategies are proposed by Porter [38].

Cost Leadership: A cost leadership strategy is based on factors that provide a cost advantage, such as efficient technologies, economies of scale, adequate logistics, etc. Nevertheless, cost leadership alone will not help to achieve a higher competitive advantage. Differentiation is still important, as the customer must perceive the product to be as good as the products of the competitors. If the organization achieves both cost leadership and differentiation, it will perform better than its competitors.

Differentiation: The organization offers superior value to its customers. Several kinds of differentiation exist depending on the context and environment of the organization. To achieve superior performance, such differentiations should allow the organization to charge a higher price, gain a higher margin, or increase the market share.

Khalifa [39] proposes seven requirements for a successful strategy:

- Focus: The organization specializes only in one segment of the market. To achieve superior performance, the chosen clients must have particular requirements that cannot be fulfilled by other competitors.
- The main objective of any strategy is to achieve and maintain a competitive advantage. Consequently, organizations should be aware of the following directives, when defining their strategy.
- Aspirational: A strategy should not be easy to achieve, as competitors will be able to copy the strategy without effort.
- Power-creating: An adequate strategy changes the power within the organization. Areas with more resources gain power over areas where resources were withdrawn.
- Directional: On the external front, a strategy should shift the power from the competitors to their organization.
- Systemic: An efficient strategy should be understood by all members of the organization and give a clear indication of where to go and where not to go. Similarly, it is not possible to implement a strategy based on individual areas, but it requires a systemic and global view.
- Intentional: Finally, a strategy must be designed, planned, and executed. A strategy does not come out of anywhere.

With the introduction of Industry 4.0, the organizational strategy also requires an update. Industry 4.0 implies changes in the whole business model of the organization and therefore involves a change in the value proposition [40]. The adoption of Industry 4.0 allows the organization to enhance its strategy toward more efficiency, especially when the strategy takes into account the aspects of client servitization, design-to-cost, an adequate supply chain, and database integration and process changes [41]. Nevertheless, organizational strategy has to rely on existing resources, capabilities, and competencies. Therefore organizations relying heavily on cost and volume strategies, as well as those that rely on fast design processes and innovative process changes benefit from the implementation of Industry 4.0 initiatives [42].

Considering the arguments given and that adequate strategic decision enhances efficiency and operational effectiveness; we propose our first hypothesis:

H1: Strategy has a positive impact on operational effectiveness.

### 2.3 Process innovation

Innovation is a key factor in fostering competitiveness [43–45]. Consequently, many organizations try to improve innovation practices by acquiring new technologies, designing new products, and/or implementing new practices and routines in their production processes. Nevertheless, many innovation initiatives fail due to different factors, such as firm-related, project-related, product-related, and market-related reasons [46].

Garcia-Quevedo et al. [47] also include financial constraints as a major reason for the failure of innovation projects. Therefore, practitioners and academics search for a formula to foster success in innovation projects. Initially, a definition of innovation has to be proposed. Such a definition is based on the author’s context and point of view, which includes social, economic, political, and cultural dimensions, among others. One of the definitions proposes that innovation is any activity of the organization to create new knowledge [48–50]. The OECD takes this definition and includes not only technological innovation but any changes that improve products, processes, and/or marketing approaches [43]. Other authors define innovation as creating competitive advantage and enhancing strategic adaptability [51, 52]. Kahn [53] defines innovation as an outcome, a process, and a mindset. From that point of view, innovation as an outcome defines what is achieved through innovation. A process includes the procedures and methods used to reach that output, and innovation as a mindset implies that every member of the organization should be aware of the benefits of innovation and foster an innovation culture.

The model developed by Tidd and Bessant [28] includes most of the findings from the literature and proposes four different dimensions of innovation: product innovation, process innovation, position innovation, and paradigm innovation. The authors postulated that paradigm innovation is the most difficult to achieve as it implies changes in the mindset of the members of the organization. On the other hand, process innovations play an equal or even more important role than product or position innovation in achieving competitive advantage. Doing something better than or different from the competitors can generate a clear competitive advantage [54, 55].

Creating something new or a radical change is not always required to be classified as innovation. Major changes require more effort and financial resources and imply bigger risks. Small changes or continuous improvement are considered innovations [53]. Similarly, Tidd and Bessant [28] classify the type of innovation, independently of its dimension, as radical or incremental. Both involve changes, but the factors of investment and risk differ substantially.

Process innovation involves changing actual production processes and methods, including how products and services are produced and delivered to the client, in search of more efficiency. Such changes could be decreased waste and cost, enhanced quality, enhanced production output, or faster processing. Generally, process innovation applies to internal areas of the organization [28, 53]. Although process innovation can be found more in mature industrial sectors and mature organizations, SMEs also require innovation in their processes to be competitive. Mature organizations try to reach more efficient ways to offer their product and services (rationalization, economies of scale, process innovation), meanwhile, new SMEs tend to be more innovative and develop new products and try to change paradigms [28, 56]. Consequently, SMEs improve process innovation through external acquisitions. Large organizations rely more on internal efforts to improve process innovation [57, 58]. Nevertheless, process innovation requires an efficient innovation and product development process in both kinds of organizations [53, 59].

Managing process innovation is not an easy task. A common vision of goals and team stability are critical factors. Additionally, adequate funding and challenging but not impossible deadlines are also significant aspects of successful process innovation initiatives. Another important feature is adequate storage and exchange of information [60, 61].

Given the previous arguments, strategy has a significant impact on how processes, structures, and routines are designed within an organization, and therefore on process innovation, we propose that:

H2: Strategy has a significant and positive impact on process innovation.

According to Tidd and Bessant [28], process innovation shifts around cost, quality, and similar factors that are parameters of operational effectiveness. Consequently, our next hypothesis proposes that

H3: Process innovation has a positive and significant impact on operational effectiveness.

### 2.4 Additive technologies

Additive technologies are a novelty in production systems. The first patent was given in 1981, but only in 2000, with the imprint of the first kidney, did the golden age of 3D printing begin [62]. After the expiration of the patent in 2005, open-source projects have fostered the implementation of additive technologies even more, not only within the academic and engineering community but by organizations as an alternative production methodology. Different industrial sectors also benefit from additive technologies, such as automotive and machinery production, health care, and even the food sector. The development of different materials, such as multi-material shape memory polymer (4D printing), 3D printing molecules, 3D printing conductive materials, 3D printing bones, tissues, and organs, eco-friendly materials for 3D Printing, or carbon nanotubes, among others, boosted the development of new manufacturing processes [63–65]. It is equally important to stress that many materials are eco-friendly, such as polylactic acid (PLA)—made from fermented sugar and polymerized to PLA. Other elements are made from recycled materials, such as acrylonitrile butadiene styrene (ABS), which can be made from recycled plastic [66–69].

In 3D-printing technology, several layers are built up, based on a CAD program, thus creating a three-dimensional model. The benefits of this technology are widely known. 3D printing allows smaller or even single lots of products at an affordable cost. Also, more complex geometrical figures are possible without the increased cost when producing with traditional machinery. The lead times on production (an important factor in the supply chain), are much shorter than with traditional processes [70].

Another important advantage is the reduction of waste in the production process. As the products are created layer by layer, the waste is almost zero, an important factor in today´s environmental discussions. Additionally, with the actual pandemic, the factor of remote printing is becoming more important. The design and instruction can be easily sent over the internet and printed anywhere a printer exists [71].

One of the main disadvantages is the actual high cost of the printed item, which makes it difficult to produce at large scales. Nevertheless, this disadvantage could be transformed into an advantage as it allows the development of products with a limited scope of differentiation between them and therefore copes perfectly with the client’s expectations and requirements [72–74].

Being part of the Industry 4.0 implies also that a strategy for implementing such new technology with success in the organization exists. Management should be aware of the benefits, risks, and difficulties of implementing 3D printing and design an adequate strategy according to the organization’s requirements [75–77]. Consequently, the fourth hypothesis states that:

H4: Strategy has a significant and positive impact on the implementation of additive technologies.

Similarly, as additive technologies are a novelty that is changing the organizational world and require adequate implementation and changes in existing processes [78, 79], we propose that:

H5: Additive technologies have a significant and positive impact on process innovation.

Furthermore, one of the main goals in introducing additive technologies into the organization is to foster the efficiency of processes, reduce waste—and therefore cost, and speed up the response to clients’ requirements for different products. Last, but not least, additive technologies allow faster responses to market changes with adequately implemented technology. Consequently, the sixth hypothesis states that:

H6: Additive technologies have a significant and positive impact on operational effectiveness.

### 2.5 Outsourcing

With the tendency to concentrate on the core activities of the organization, outsourcing strategy emerges as an alternative to improve production. Nevertheless, knowing what the core activities of the organization are, has a significant impact on the design of the organizational strategy. Outsourcing refers to the transfer of activities to external parties. Although both internal and external activities create value for the client, sometimes it is more efficient for the organization to externalize some non-core activities to parties who can provide the required services faster and cheaper [80–82].

Initially, most of the outsourcing practices affected third-party logistics (3PL) as a strategy to reduce cost and enhance agility [83, 84]. Nevertheless, with globalization, outsourcing has become an alternative for multiple areas of the organization. Customer services, accounting, taxes, programming, human resources, or providing part of a product are only a few of the organizational activities that are candidates for outsourcing [85, 86].

The main goal of outsourcing any activity of the organization is to reduce the cost to achieve a competitive advantage [87, 88]. Other reasons to choose to outsource are changes in organizational strategy, more efficient management of the supply chain, enhancement of the quality of the products, incrementing the strength of external networks, business process-reengineering, capital investment reduction, flexibility enhancement, or knowledge exchange [86, 89–91].

Outsourcing is a strategic decision made by the management, with its known uncertainties [89, 92]. It is expected that the strategic decisions related to technological factors that boost outsourcing processes will improve the sustainable competitive advantage [91, 93, 94]. Consequently, our seventh and eighth hypotheses state that:

H7: Strategy has a positive and significant impact on outsourcing.
H8: Outsourcing has a positive and significant impact on operational effectiveness.

Nevertheless, outsourcing activities implies changes in the processes of the organization. As different services are now provided by external parties, the actual processes need to be changed and coordination efforts will increase. It is expected that the cost-saving (or other benefits) compensates for the increase in coordination costs [95–97]. Process innovation means changes in existing practices and the consequences of those enhancements lead to outsourcing certain activities. Consequently, the outsourcing activities also depend on process changes planned by the organization [98, 99]. Therefore, our ninth hypothesis states that:

H9: Process innovation has a positive and significant impact on outsourcing activities.

Finally, when clients want to make small changes to existing products, or when a part of a machine has broken and the replacement is no longer available, 3D printing becomes the appropriate solution. The production of unique replacements is not profitable for the manufacturer and, consequently, a completely new service, the 3D printing industry, emerged as a solution [100]. Organizations can now abstain from having a large inventory for certain product parts and have instead an inventory of digital product designs, ready to be printed when required. As owning a 3D printer is not always cost-efficient, the files with the item specifications can be easily sent through the internet to an external service provider, thus taking advantage of the outsourced activity [101]. Although organizations recognize the benefits of 3D printing, reasons such as high printer acquisition cost, lack of experience with such a new technology, the technical limitations of a printer (materials, dimensions), or maintenance cost, foster the use of external third parties [100–102]. Therefore, our tenth and last hypothesis state:

H10: Additive technologies have a significant and positive impact on outsourcing.

**Fig 1:**
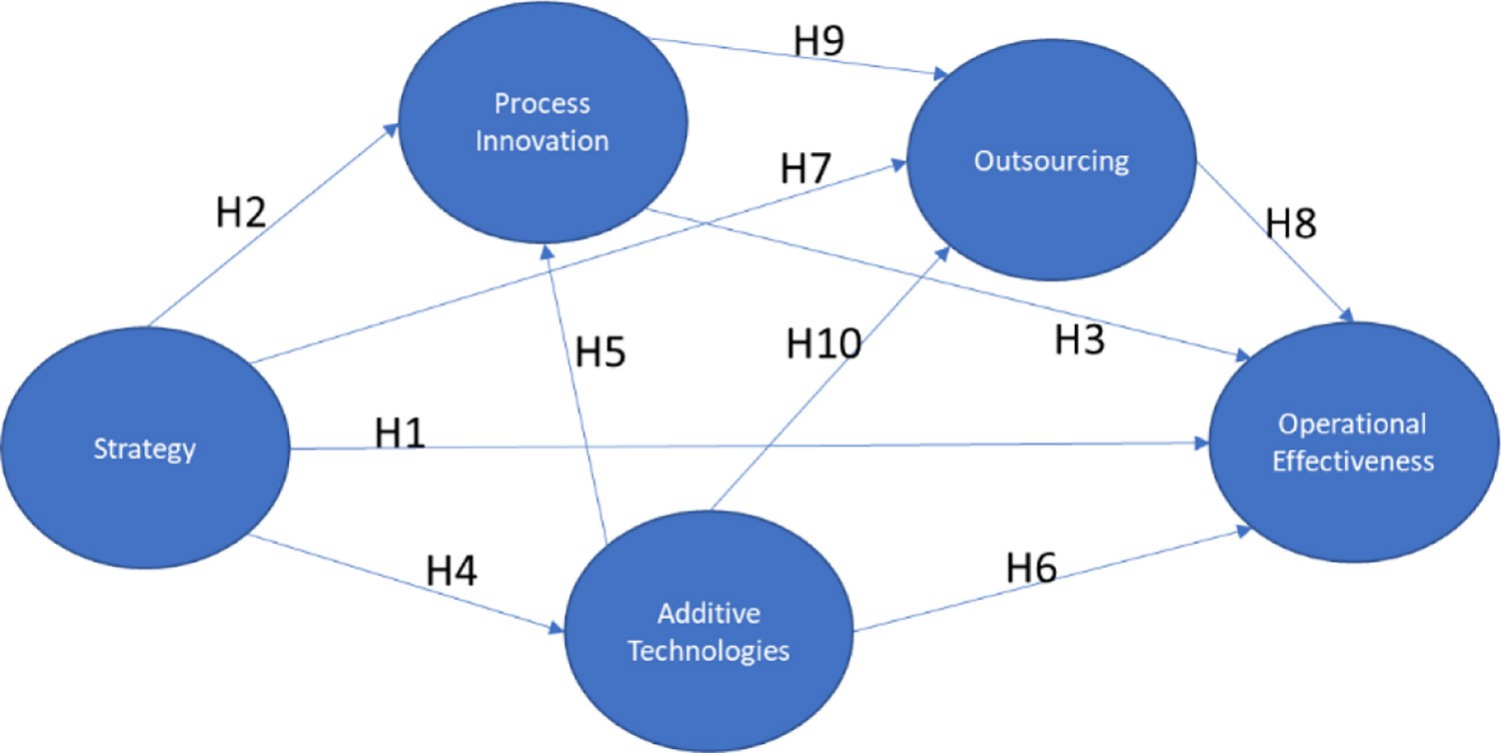
Theoretical model. Source: By the authors.

Fig 1 shows the theoretical model with the hypothesis.

## 3. Methodology

**Fig 2:**
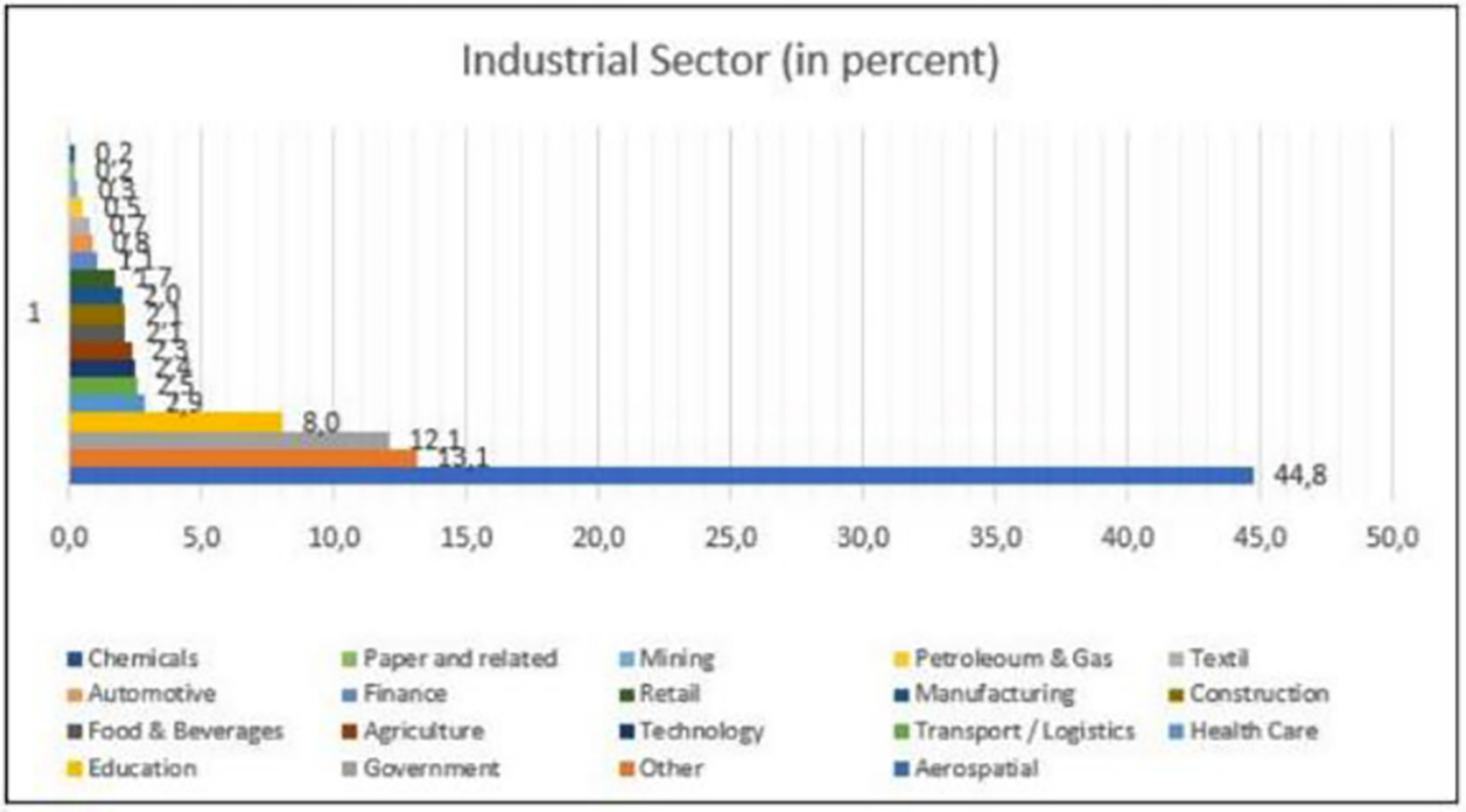
Industrial sector of the respo 1. Source: By the authors 1.

**Fig 3:**
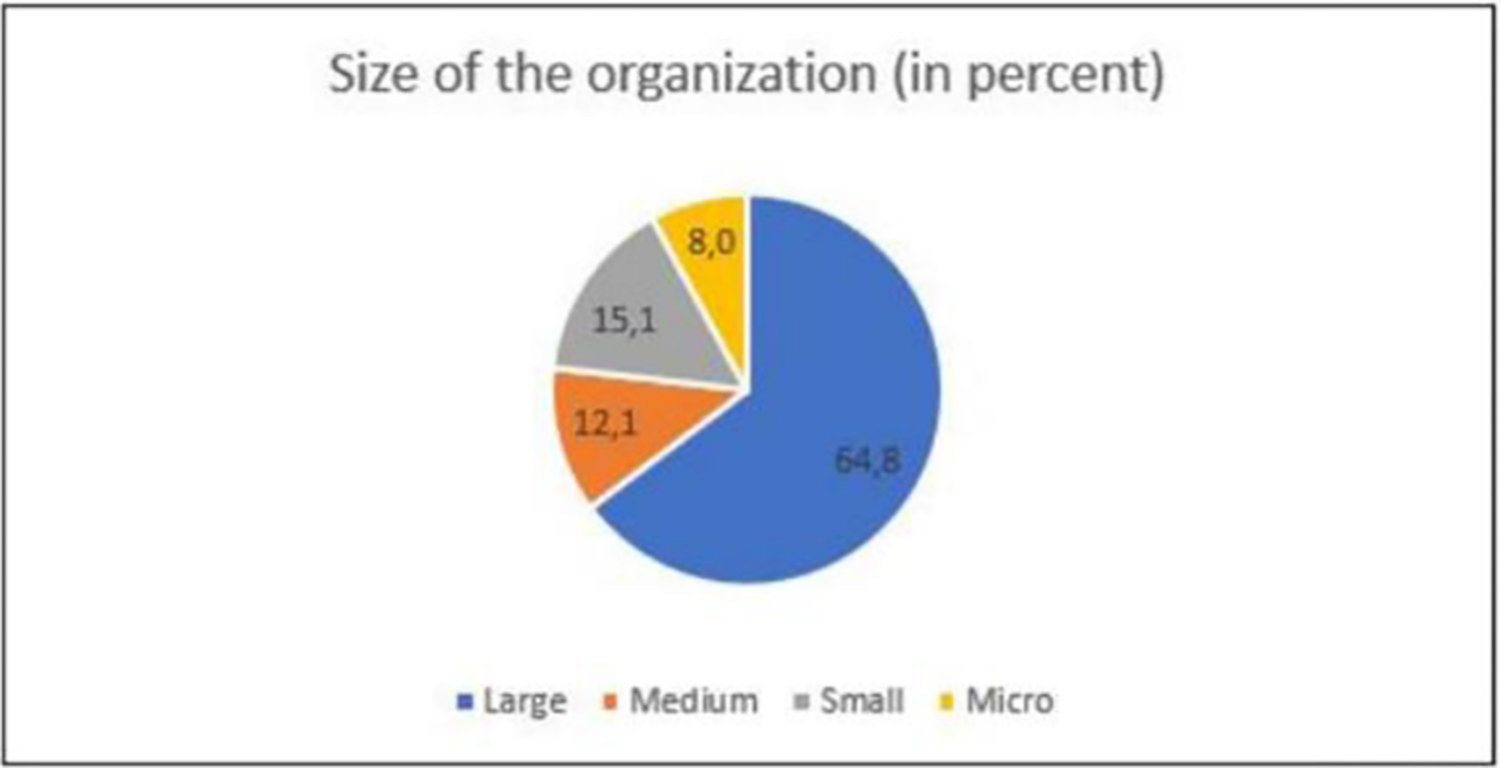
Size of the respondent´s organization. Source: By the authors.

This research is a confirmatory study, as the aim is to sustain pre-specified relationships [103] borrowed from the models of Tidd and Bessant [28], Santa et al. [14], and Ferrer et al. [104]. A self-administered questionnaire was developed according to the guidelines established by Hair et al. [105] and sent to the participants. The survey consisted of a first section on the demographics, followed by questions related to the conceptualized set of variables necessary to build the desired model. The questionnaire uses a five-point-Likert-type scale, ranging from strongly agree to strongly disagree. Of 994 responses, 946 were considered usable (95%) and 48 were discarded (due to missing or inconsistent data). To test the hypotheses, descriptive and inferential statistical analysis was used to analyze the collected data. Figs 2 and 3 show the demographic data of the respondents of the survey.

It is important to stress that the respondents of large organizations accounted for 64% of all answers. Large organizations are more prompt to use new and innovative technologies, such as additive technologies, and leverage the associated benefits [106–108].

The average mean values of the statements’ ratings were used to build the variables that made up the structural equation model. This methodology was chosen as it fits the requirements of this research and allows the analysis of latent variables and their relationships and the required sample is met by the collected data [109].

Both SPSS and Amos were used to undertake the multivariant analysis. Factor loadings were estimated, the items loaded on only one construct (i.e., no cross-loading) and latent constructs were correlated. The internal consistency was assessed by Cronbach’s alpha coefficient and the items-to-total correlation. All values were above 0.7, the cut-off level for basic research,as shown in Table 1 [110, 111].

**Table 1:** Validation of the final measurement model.

		USRW	SRW	Robust t-value	P	LA	CA	CR	AVE
Strategy	ES5	1	0,891			0,822	0,920	0,958	0,680
	ES4	0,938	0,842	36,038	***				
	ES3	0,869	0,758	29,619	***				
	ES1	0,903	0,712	26,741	***				
	ES6	0,966	0,873	38,913	***				
	ES7	0,949	0,855	37,209	***				
Process Innovation	IP1	1	0,833			0,875	0,930	0,961	0,767
	IP2	1,05	0,858	33,065	***				
	IP3	1,038	0,91	36,539	***				
	IP4	1,022	0,899	35,773	***				
Operational effectiveness	EO10	0,981	0,866	34,858	***	0,824	0,940	0,969	0,683
	EO9	0,955	0,765	28,406	***				
	EO8	1,043	0,885	36,259	***				
	EO7	0,937	0,779	29,202	***				
	EO5	0,959	0,727	26,32	***				
	EO4	1,007	0,857	34,141	***				
	EO3	1,028	0,87	35,051	***				
	EO2	1	0,846						
Industry 4.0	IND7	1	0,796			0,841	0,950	0,973	0,709
	IND8	1,023	0,837	37,46	***				
	IND9	1,06	0,8	27,893	***				
	IND11	1,087	0,892	32,492	***				
	IND14	1,022	0,841	29,825	***				
	IND15	1,038	0,861	30,845	***				
	IND16	1,053	0,844	30,003	***				
	IND17	1,099	0,859	30,745	***				
Outsourcing	OUT1	1	0,764			0,722	0,900	0,929	0,523
	OUT2	0,992	0,783	29,5	***				
	OUT3	0,984	0,687	21,297	***				
	OUT4	0,901	0,739	23,104	***				
	OUT5	0,933	0,724	22,587	***				
	OUT7	0,952	0,706	21,929	***				
	OUT9	0,887	0,652	20,045	***				

USRW: Unstandarized regression weigth / SRW: Standarized regression weight / LA: Loadings average / CA: Cronbachs Alpha

CR: Composite reliability / AVE: Average variance extracted

Source: By the authors

Confirmatory factor analysis (CFA) was used to measure the relationships between observed and continuous latent variables, and to determine the model’s overall fit [105, 112, 113].

The model shows 561 distinct sample moments with 82 distinct parameters to be estimated. The Chi-square equals 1832.757 with 479 degrees of freedom. The value of CMIN/DF of 3.83 and a 0.000 probability level suggested that the model has a good fit. Wheaton et al. [114] suggested a ratio of five or less as acceptable, other authors recommend values between 2 and 5 [112, 115]. The values of CFI (0.953), NFI (0.938), RFI (0.931), IFI (0.953) and TLI (0.948) above 0.9 support the model [115–118]. The value of the root mean square error of approximation (RMSEA) of 0.055 was good, close to 0.05 [116]. Consequently, the baseline comparison indices suggest that the hypothesized model fits the observed variance-covariance matrix well relative to the null or independence model (Tables 2 and 3).

**Table 2:** Discriminant validity 1.

	1	2	3	4	5
Strategy	0,824	0,833	0,863	0,72	0,771
Process Innovation		0,876	0,858	0,724	0,834
Operational effectiveness			0,826	0,768	0,827
Industry 4.0				0,842	0,792
Outsourcing					0,723

Source: By the authors

**Table 3:** Baseline comparisons 1.

Model	NFI Delta1	RFI rho1	IFI Delta2	TLI rho2	CFI
Default model	.938	.931	.953	.948	.953
Saturated model	1.000		1.000		1.000
Independence model	.000	.000	.000	.000	.000

Source: By the authors

## 4. Results

**Fig 4:**
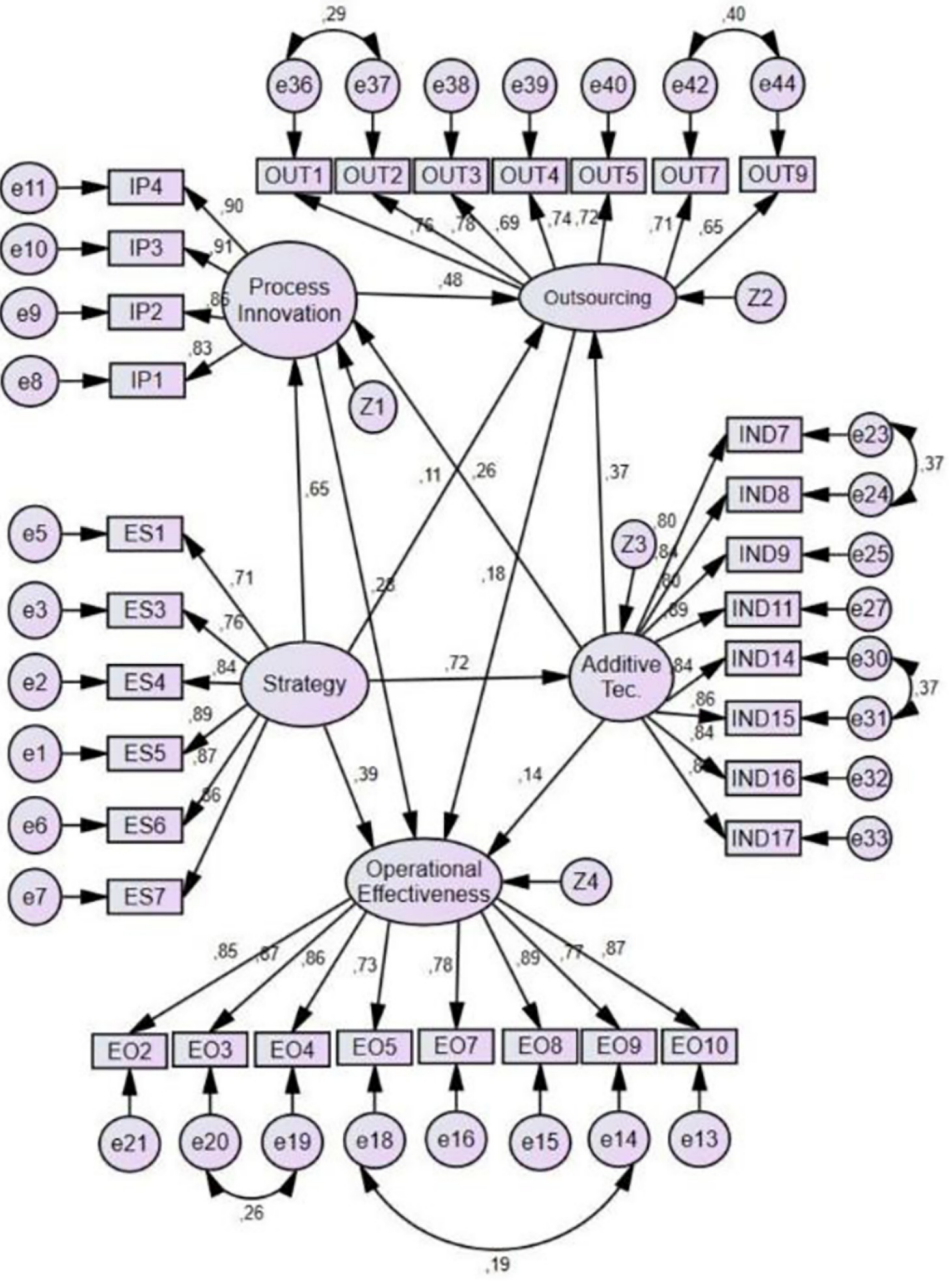
Model results. Source: By the authors.

The findings from SEM shown in Fig 4 and Table 4 show a strong relationship between strategy and process innovation (β = 0.62, p < 0.001), and between additive technologies and strategy (β = 0.33, p < 0.001). The results also show a medium relationship between process innovation and outsourcing (β = 0.43), p < 0.001), additive technologies and outsourcing (β = 0.36, p < 0.001), strategy and operational effectiveness (β = 0.34, p < 0.001), process innovation and operational effectiveness (β = 0.27, p < 0.001), thus not rejecting Hypothesis 1, 2,3, 4, 9, and 10. Between additive technologies and operational effectiveness (β = 0.14, p < 0.001), process innovation and additive technologies (β = 0.27, p < 0.001), and between outsourcing and operational effectiveness (β = 0.18, p < 0.001) there is only a low relationship, but nevertheless, hypothesis 5, 6 and 8 were not rejected. The relationship between strategy and outsourcing was not supported (H7).

**Table 4:** Regression weights: (Group number 1—Default model).

			Est.	S.E.	C.R.	P	
Process_Innovation	<-	Strategy	.618	.034	18.321	***	H2-not rejected
Additive_Tec.	<-	Strategy	0.633	0.029	21.977	***	H4-not rejected
Process_Innovation	<-	Additive_Tec.	0.273	0.035	7.856	***	H5-not rejected
Outsourcing	<-	Strategy	0.092	0.04	2.328	0.02	H7-rejected
Outsourcing	<-	Process_Innovation	0.431	0.045	9.641	***	H9-not rejected
Outsourcing	<-	Additive_Tec.	0.364	0.036	10.202	***	H10-not rejected
Operational_Effectiveness	<-	Strategy	0.347	0.033	10.509	***	H1-not rejected
Operational_Effectiveness	<-	Process_Innovation	0.278	0.041	6.744	***	H3-not rejected
Operational_Effectiveness	<-	Additive_Tec.	0.143	0.032	4.468	***	H6-not rejected
Operational_Effectiveness	<-	Outsourcing	0.179	0.046	3.855	***	H8-not rejected

Source: By the authors

## 5. Discussion

The results of this study reveal that Colombian organizations are not leveraging the benefits of Industry 4.0, such as process innovation and additive technologies. Colombia has high potential because one of the main benefits of Industry 4.0 is the decentralized manufacturing and personalization of production, but other factors prevent successful Industry 4.0 initiatives. The main factor seems to be the digital gap. Access to digital services is very different between urban and rural areas. Urban areas have a better quality of digital services at a lower cost. Additionally, access for small and medium enterprises is far more difficult and costly than for large organizations [12]. Consequently, small and medium organizations not only have higher costs than large companies but also lose competitiveness against the large local competitors and foreign organizations.

Another issue seems to be the general lack of trust. Colombia is a country with a low level of trust between government, citizens, and organizations [119–121]. In particular, the lack of trust between the organization and contractors outside the organization constitutes an obstacle to leveraging benefits from outsourcing activities [122]. The mistrust between the different components of society not only affects the implementation of technological innovations but also prevents the collaboration between them and generates inefficiencies in the use of resources. That includes the exchange of information and the awareness of the benefits of adequate implementation of Industry 4.0 [12, 123–125].

Another factor of lesser importance is the lack of skills in digital business and managerial knowledge of Colombian workers. Although in Colombia there are many high-ranked and internationally accredited educational institutions, more focused development of human resources in digital capabilities and competencies is required [12, 126, 127].

Considering the hindering factors, it is not surprising that additive technologies and process innovation only add a very low level to organizational performance. Although the management is aware of the benefits of Industry 4.0 and designs strategies to implement such initiatives, it is not enough. The fact that the strategies do not have an impact on outsourcing confirms the view of mistrust between the actors, even though this study also shows that changes in processes and implementing additive technologies have an impact on outsourcing activities. Surprisingly, it seems that the outsourcing of activities has a small, but measurable effect on operational effectiveness. We suggest that such effects are consequent to some personal relationship, and therefore a greater amount of trust, between the outsourcing organization and the executant of the outsourced activities and not as a result of a planned organizational strategy.

## 6. Conclusions

As the results of this research show that Colombian organizations are not fully exploiting the possibilities of the Industry 4.0 initiatives, the government, academia, and organizations should invest in initiatives that create trust, enhance digital skills, and close the digital gap.

Academia should implement courses in digital knowledge, such as big data, cloud computing, etc. It also should encourage teamwork and the exchange of information between the students. Such initiatives should help to understand the concept of Industry 4.0 and the importance of teamwork in implementing technological innovations and the benefits of collaboration. Additionally, such training should enhance the transversal exchange of knowledge between different faculties, as Industry 4.0 requires not only technical skills but also managerial, social, and methodological competencies.

The government should enhance digital infrastructure and promote trust. The acceptance of new technologies depends on such initiatives. The government should also encourage educational institutions to develop programs in digital education starting from primary school and encourage studies in a digital career. Projects that generate trust between the different aspects are urgently required, not only to support the acceptance of technological initiatives, such as e-government but also to facilitate the interaction between the different members of Colombian society.

Organizations should foster the interaction between their workforce, to enhance collaboration and information exchange, not only internally, but also with external actors. Similarly, digital education should be one of the main topics in backing advanced studies of their workforce and the support of educational institutions on all levels in digital education is vital.

Without such initiatives, the implementation of the Industry 4.0 concept will be very slow and might even come to a halt. The consequences are a decrease in competitiveness with the consequent slower advancement of social, political, and economic dimensions in society.

## 7. Limitations and further research

This article has several limitations. First, the study used a convenience sample, with respondents chosen based on their operations and practices, knowledge, experience, expertise, and tenure in the management and implementation of Industry 4.0 activities. Second, the sample size is limited when compared to other studies in industrialized countries. It should also be noted that about half of Colombia’s labor force is informal, and the study is based on responses from members of formal organizations. Additionally, Colombia is also a country with a high level of mistrust. Given these considerations, generalizability across all industrial sectors appears to be a stretch. Nonetheless, the findings reveal information that justifies a broader, quantitative study. Further research might look at why Colombia is one of the most distrusted countries in the world and what can be done to change it.
